# Mapping Knowledge Structure and Themes Trends of Post-operative Rehabilitation of Hip Fractures in the Elderly: A Bibliometrics and Visualization Study

**DOI:** 10.3389/fsurg.2022.881555

**Published:** 2022-05-03

**Authors:** Liuchao Hu, Qiushi Wei, Ziheng Luo, Bin Wang, Zhifang Wu, Mincong He, Xiaoming He, Yiwen Luo

**Affiliations:** ^1^The Third Affiliated Hospital, Guangzhou University of Chinese Medicine, Guangzhou, China; ^2^Guangdong Research Institute for Orthopedics and Traumatology of Chinese Medicine, Guangzhou, China

**Keywords:** hip fracture, postoperative rehabilitation, bibliometric analysis, research trends, hotspots

## Abstract

**Background:**

Hip fractures are a common type of fracture in the elderly and are characterized by many complications and high mortality. Many topics concerning postoperative rehabilitation of hip fracture in elderly people still remain controversial, and the global research trend in this field has not yet been well studied. The aim of the present study was to illustrate the overall knowledge structure, development trends, and research hot spots of postoperative rehabilitation of hip fracture in elderly people.

**Methods:**

Articles and reviews regarding postoperative rehabilitation of hip fracture in elderly people from 2001 to 2021 were identified from the Web of Science database. An online bibliometric platform, CiteSpace, and VOSviewer software were used to generate visualization knowledge maps, including annual trends of publications, contributions of countries, institutions, authors, funding agencies, and journals, and clustering of keywords.

**Results:**

A total of 1,724 publications were identified from Web of Science Core Collection (WoSCC). In the past 20 years, the number of published studies on the rehabilitation of hip fracture in the elderly has exhibited an overall upward trend. The USA was the leading contributor in this field, with the largest number of publications (354, 20.65%) and the most citations (13,786 times). The international cooperation map among relevant countries/regions indicated that the USA collaborated most closely with Canada and China. The University of Maryland and Professor Marcantonio were the most prolific institution and influential author, respectively. *Injury: International Journal of the Care of the Injured* was the most productive journal concerning the research of postoperative rehabilitation of hip fracture in elderly people. The keyword co-occurrence analysis identified six clusters: quality of life study, rehabilitation and outcomes study, cognitive impairment study, operative approaches study, mortality study, and osteoporosis study.

**Conclusions:**

There will be an increasing number of publications on the research of postoperative rehabilitation of hip fracture in elderly people, and the United States will stay ahead in this field. Our findings could offer practical sources for scholars to understand the current status and trend of studies on rehabilitation of hip fracture in the elderly and provide references and suggestions for the development of related research in future.

## Background

Hip fractures are a common type of fracture in the elderly and are characterized by many complications and high mortality (1). These patients often have osteoporosis and complex conditions, making it difficult for orthopedists to treat them. Therefore, it is known as the last fracture of life. With advances in medical technology, surgical treatment is generally recommended for hip fracture in the elderly if the condition permits (2). Its purpose is to get patients out of bed early to prevent bed complications such as deep vein thrombosis, hypostatic pneumonia, bedsore, cerebral infarction, and pulmonary embolism. Following hip fracture surgery, postoperative patient rehabilitation is important to achieve the optimal level of hip function and mobility. Poor postoperative recovery not only seriously affects the quality of life, but also brings heavy economic burden to patients (3). In recent years, several protocols have been developed to improve for recovery following hip fracture surgery (4, 5). For example, multidisciplinary rehabilitation decreased the likelihood that patients with hip fracture would have a poor outcome, including death or admission to a nursing home. However, there are some challenges in clinical implementation of these protocols (6). Furthermore, home-based rehabilitation after hip fracture surgery seems to be a good alternative to classic inpatient rehabilitation. A new study shows that patients with more complex medical conditions who underwent inpatient rehabilitation had similar rehabilitation outcomes as patients with less medically complex conditions who underwent home-based rehabilitation (7). In addition, adequate nutritional supplementation may be useful in decreasing complications and shortening the length of stay both in the hospital and during rehabilitation in elderly patients with hip fractures (8). Despite much new understanding in this area, there is no consensus regarding standardized methods for postoperative rehabilitation. Therefore, research in this field continues and is important for the development of postoperative rehabilitation of hip fracture with improvement in clinical outcome.

Literature is an important way to explore a certain research field and an important part of global scientific research. Bibliometric analysis can provide information based on a literature database and bibliometric characteristics for qualitative and quantitative assessment of research activity trends over time. It provides a way to capture developments in a field and predict future research directions (9, 10).

Worldwide, many authors have published clinically relevant research findings on hip fracture, including studies on surgical treatment of choice, risk factors affecting prognosis, perioperative management, rehabilitation training, anti-osteoporosis treatment and management measures for patients after discharge (11–15) (González-Quevedo et al., 2020). Clinical research in this area is growing, and bibliometric analysis can provide direction for clinical researchers and improve the understanding of research trends, journal selection, and key topics. However, there are few bibliometric studies on the postoperative rehabilitation of elderly patients with hip fracture.

Therefore, this bibliometric study aimed to analyze the literature on rehabilitation of hip fracture in the elderly from 2001 to 2021 to understand the current status and trend in studies on rehabilitation of hip fracture in the elderly and provide references and present suggestions for the development of related research in future.

## Materials and Methods

### Data Source

The data for this study were collected from the Science Citation Index Expanded (SCI-Expanded) of the Web of Science Core Collection (WoSCC). Web of Science is an internationally recognized database reflecting the level of scientific research. It includes numerous influential high-quality journals in the world. It is also one of the most frequently used databases in previous bibliometric studies (16).

### Retrieval Strategies

**Figure 1:**
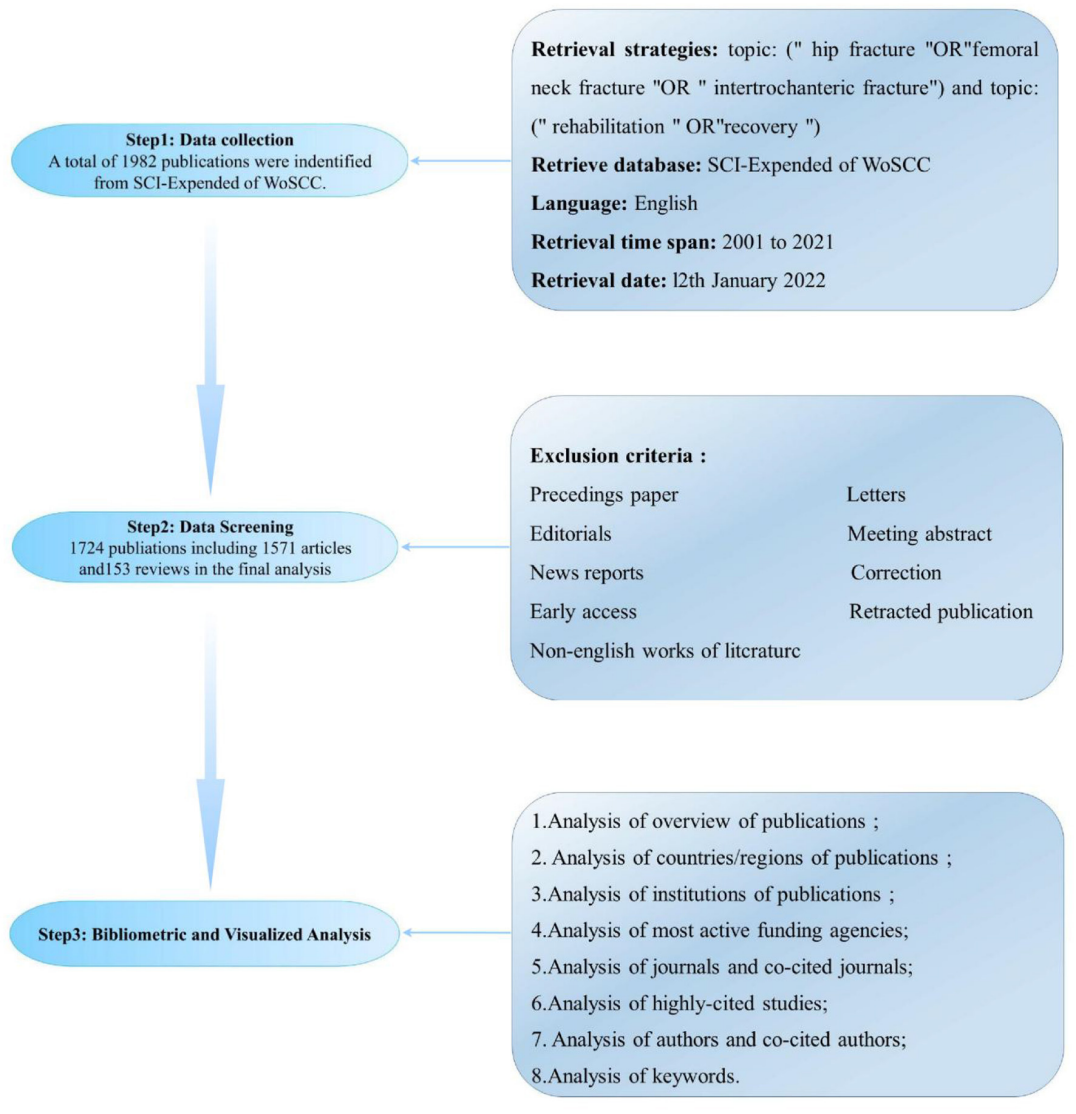
Flowchart of literature selection and data analysis.

The retrieval strategy was as follows (Figure 1): TI: “hip fracture” OR “femoral neck fracture”, OR “intertrochanteric fracture”; topic: “rehabilitation” OR “recovery”; language: English. The publication types were limited to original articles and reviews, excluding papers from proceedings, letters, editorials, meeting abstracts, news reports, editorials, corrections, early access, retracted publications, and non-English works of literature. The search was conducted for publications between 2001 and 2021. The date of the retrieval was 12 January 2022.

### Data Extraction and Descriptive Analysis

The full record of each document, including title, date of publication, author, countries/regions, published journal name, keywords, and abstract, was downloaded from the WoSCC as a txt file and imported into Microsoft Excel 2019 for further data processing and graph plotting. Two authors independently sifted and extracted data for entry and collection. Any differences were resolved by consensus through discussion. Data extracted from the selected articles include the general information about the annual number of publications, citation frequency, average citations per item (ACI), original countries and institutions, authors, journals, funding agencies, and H-index.

### Bibliometric and Visualized Analysis

VOSviewer, CiteSpace (Chaomei Chen, Drexel University, USA) and an online analysis platform were used to perform this bibliometric study. VOSviewer is developed by Van Eck and Waltman of the Center for Science and Technology Studies (CWTS) of Leiden University in the Netherlands. It is a document knowledge unit visualization software based on similarity visualization (VOS) technology, which has unique advantages in mapping the display of the knowledge domain. VOSviewer is used to visualize the coauthorship of countries, authors and organizations, the co-citation of sources, and the co-occurrence of keywords. In the network visualization map created by VOSviewer, different nodes represent various parameters, such as countries, journals, and keywords. The size of nodes in the map is proportional to the number of publications, references, or occurrences. Total link strength (TLS) represents the connection strength between the nodule and other nodes (17).

CiteSpace is a visual analysis software that focuses on analyzing the trends and dynamic changes in scientific research literature and identifies key points in a given field. Betweenness centrality (BC) is an important parameter of centrality that can assess the scientific importance of the nodes in a network, where nodes with high BC value (≥0.1) are usually indicated by purple rings. For the dual-map overlay of journals, the labels represented different research subjects covered by the relevant journals. The left side of the map displayed citing journals, while the right side displayed cited journals. Different colors and widths of lines originating from the citing map and ending at the cited map indicate the paths of the citation links (18). In this study, the default parameters are used in CiteSpace and VOSviewer.

## Results

### Analysis of the Overview of Publications

**Figure 2:**
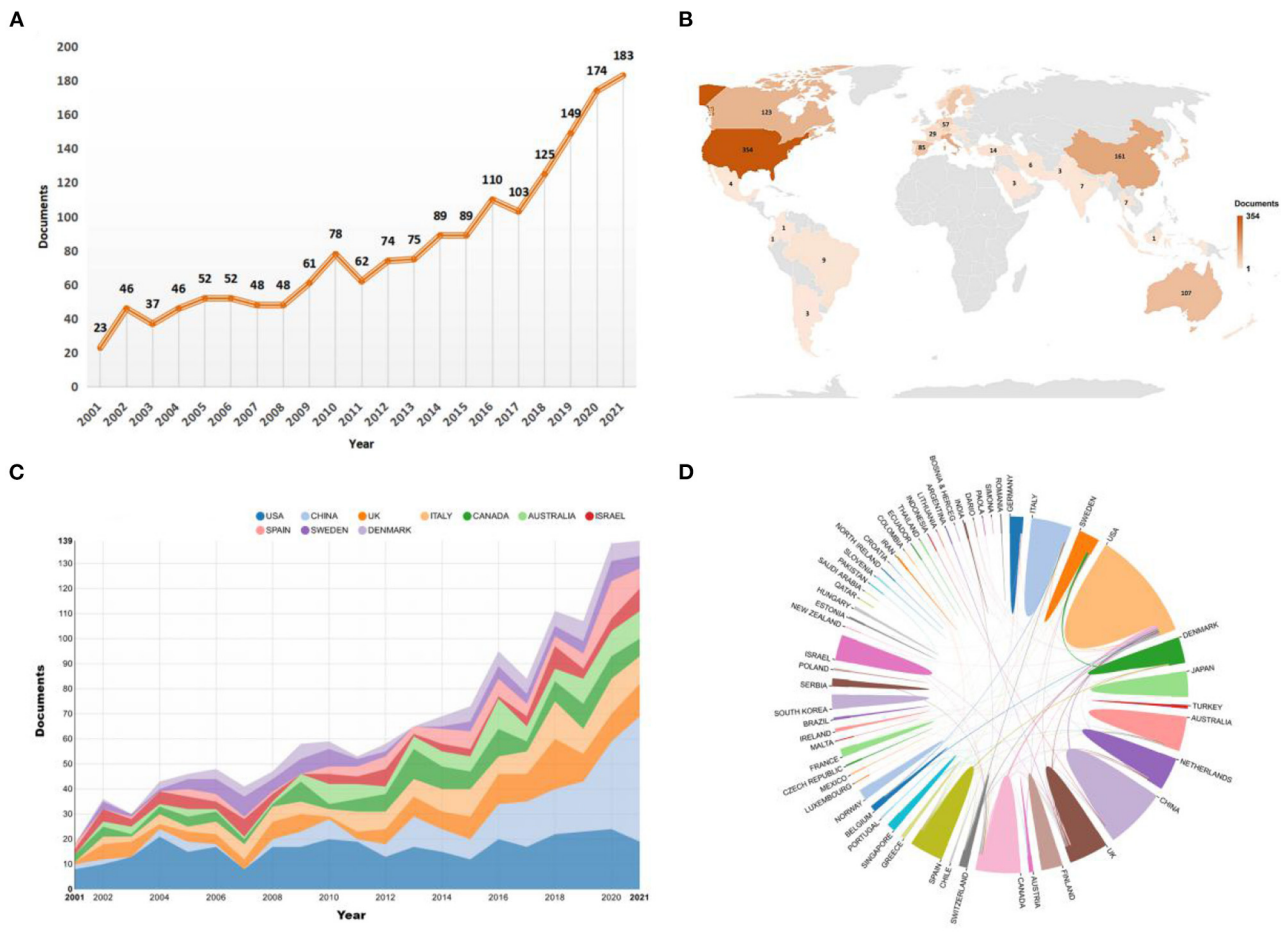
**(A)** The number of annual publications on rehabilitation of hip fracture from 2001 to 2021. **(B)** A world map depicting the contribution of each country based on publication counts. **(C)** The annual number of publications in the top 10 most productive countries from 2001 to 2021. **(D)** International collaboration analysis among different countries/regions. **(C,D)** was generated from an online analysis platform (https://bibliometric.com).

A total of 1,724 publications, including 1,571 articles and 153 reviews, were extracted from WoSCC. In the past 20 years, the number of published studies on the rehabilitation of hip fracture in the elderly has exhibited an overall upward trend. Since 2016, more than 100 papers have been published per year with a peak in 2021, with 183 papers (Figure 2A). Research in this area is expected to continue to grow in future.

### Analysis of Countries/Regions of Publications

The top 10 countries/regions of publication of these papers are noted in Supplementary Table S1. The USA was the foremost productive country, with 354 papers published (20.65%), followed by China (161, 9.39%) and Italy (146, 8.52%). The top countries accounted for 80% of the total publications. Research from the USA was cited 13,786 times, ranked first of all the countries, followed by Canada (3,751 times) and England (3,715 times). The geographical distribution of global publications is shown on a map in Figure 2B. Most countries except Africa and Eastern Europe have published relevant literature. A transformative trend in the annual publication counts of the top 10 countries/regions from 2001 to 2021 is illustrated in Figure 2C. China has published the largest number of documents in the past 5 years. As shown in Figure 2D, the international cooperation map among relevant countries/regions indicated that the USA collaborated most closely with Canada and China.

### Analysis of Institutions of Publications

**Figure 3:**
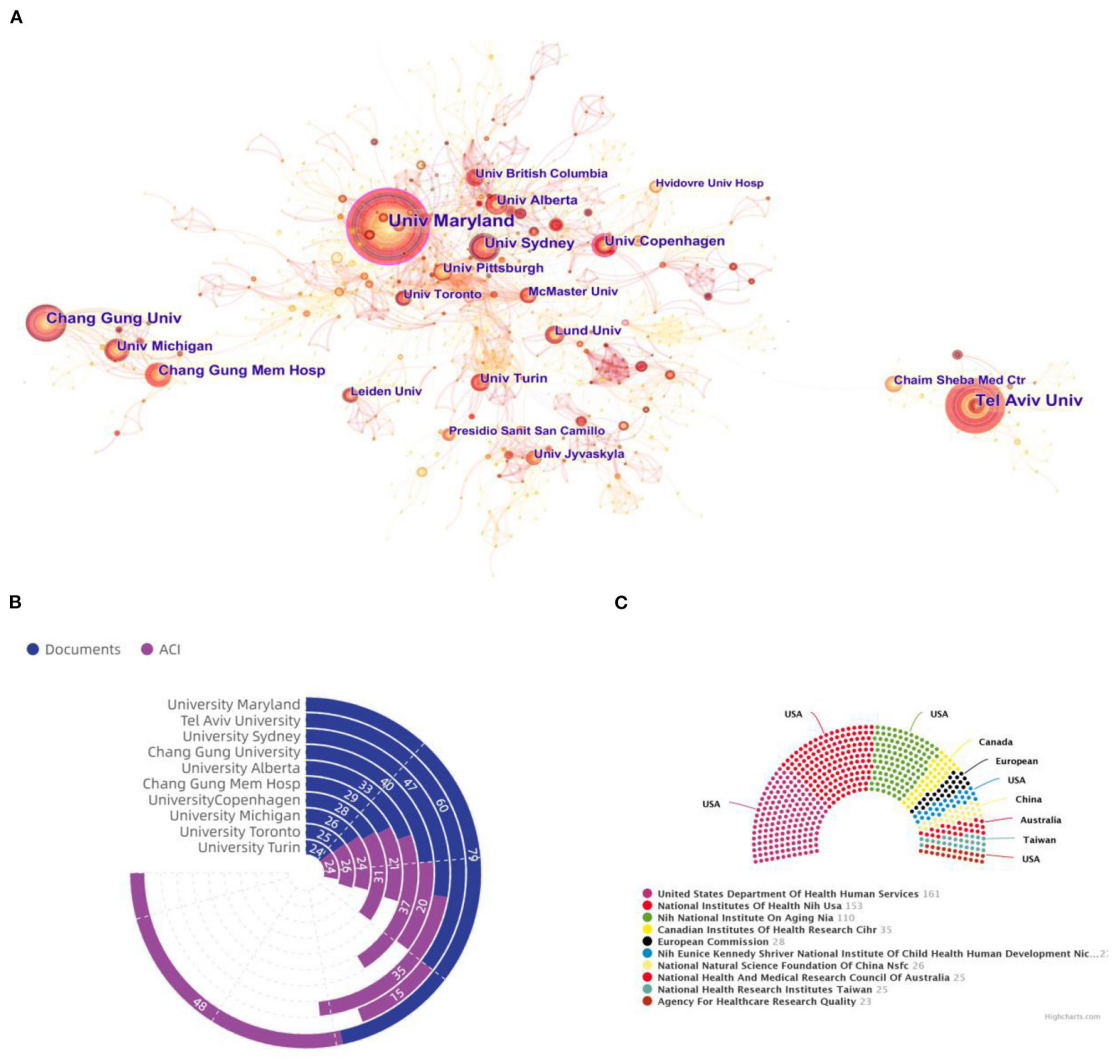
**(A)** Cooperation visualization map of the rehabilitation of hip fracture research network was generated by CiteSpace. **(B)** The document counts and ACI of the top 10 most institutions. **(C)** The top 10 most active funding agencies for research on rehabilitation of hip fracture.

For the analysis of institutions, the rough estimate is that more than 2,255 institutions have made contributions to this field. As shown in Figure 3A, a cooperation visualization map of the rehabilitation of hip fracture research network was generated by CiteSpace. The interinstitutional collaboration was relatively low and mainly conducted in European and American institutions. The University of Maryland and the University of Copenhagen occupied the center location of the collaboration network and were the only institutions with a value of BC >0.1. The ring bar diagram of Figure 3B shows the document counts and ACI of the top 10 most prolific institutions in detail. Among these, the most productive institutions on hip fracture were the University of Maryland (79 documents), followed by Tel Aviv University (60 documents), and the University of Sydney (47 documents). The top three institutions with the highest ACI values were the University of Maryland (48.19 times), the University of Alberta (36.82 times), and the University of Sydney (35.11 times).

### Analysis of the Most Active Funding Agencies

The economic foundation plays an important role in scientific development. In view of this, a brief summary of the top 10 most active funding agencies and sponsors in this area is provided in Figure 3C. Among these, there were five American institutions. The remaining were from the European Union, China, Canada, Taiwan, and Australia. The top three most active funding agencies were the United States Department of Health and Human Services (161 studies), the National Institutes of Health (NIH), USA (153 studies), and the National Institute on Aging (NIA) (110 studies). As is evident from these results, in addition to the well-established institutions, the USA maintained its leading position in the domain of the research on rehabilitation of hip fracture and cannot be separated from the support of adequate funding.

### Analysis of Journals and Co-cited Journals

**Figure 4:**
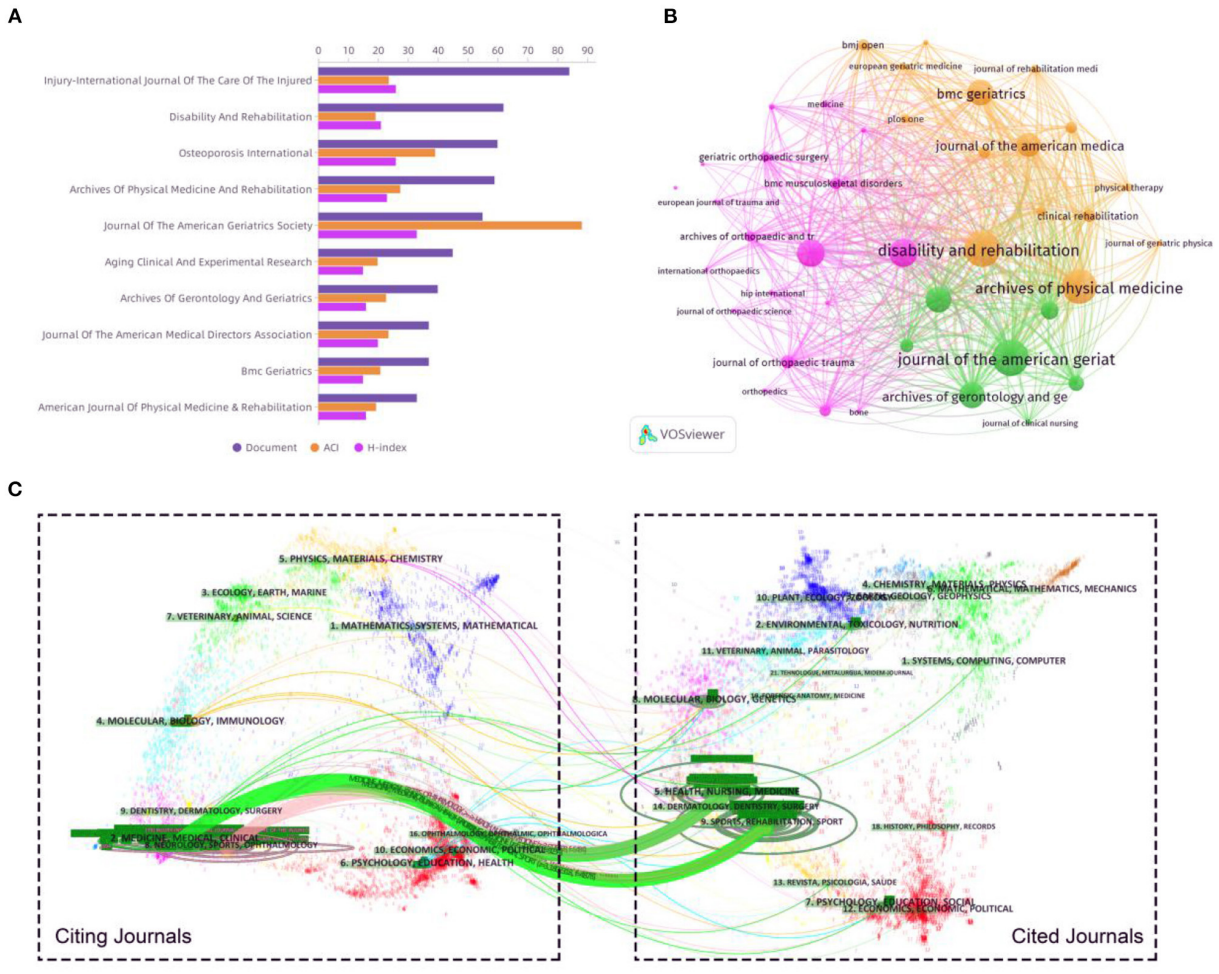
**(A)** The document counts, H-index, and ACI of the top 10 journals. **(B)** Network visualization map of journal co-citation analysis based on VOSviewer software. **(C)** The dual-map overlay of academic journals on rehabilitation of hip fracture in the elderly (generated by CiteSpace software).

A total of 362 journals have emerged recently in this research field. The 10 journals with the most publications on rehabilitation of hip fracture in the elderly are listed in Table 1. *Injury: International Journal of the Care of the Injured* published the most articles/reviews (84 papers). *Disability and Rehabilitation* ranked second, with 62 publications, followed by *Osteoporosis International* (60 papers). According to the JCR 2020 standards, the top 10 most active journals were classified as Q1 in 5, Q2 in 2, and Q3 in 3. The highest Impact Factor (IF) belongs to the *Journal of the American Geriatrics Society* (5,562), followed by the *Journal of the American Medical Directors Association* (4,669) and *Osteoporosis International* (4,507). The IF of the top 10 journals ranged from 2,586 to 5,562. Figure 4A demonstrates the document counts, H-index, and ACI of the top 10 most prolific institutions in detail. VOSviewer software was used to analyze the co-citation of journals. As shown in Figure 4B, 41 journals with at least 10 citations were included. The top three journals with the largest TLS were listed as follows: *Disability and Rehabilitation, Journal of the American Geriatrics Society*, and *Archives of Physical Medicine and Rehabilitation*. A dual-map overlay of the journals on rehabilitation of hip fracture in the elderly is shown in Figure 4C. As seen from this figure, there were four main citation paths in the dual map (green paths and pink paths).

**Table 1:** Top 10 journals in the study on rehabilitation of hip fracture in the elderly ranked by the publication number.

**Journal title**	**Documents**	**ACI**	**H-index**	**IF (2020)**	**JCR (2020)**
Injury-International Journal of The Care of The Injured	84	23.63	26	2.586	Q3
Disability and Rehabilitation	62	19.21	21	3.033	Q1
Osteoporosis International	60	39.15	26	4.507	Q2
Archives of Physical Medicine and Rehabilitation	59	27.47	23	3.966	Q1
Journal of the American Geriatrics Society	55	88.22	33	5.562	Q1
Aging Clinical and Experimental Research	45	19.89	15	3.636	Q3
Archives of Gerontology and Geriatrics	40	22.73	16	3.25	Q3
Journal of the American Medical Directors Association	37	23.51	20	4.669	Q1
BMC Geriatrics	37	20.81	15	3.921	Q2
American Journal of Physical Medicine & Rehabilitation	33	19.39	16	2.159	Q3

*ACI, average citations per item*.

### Analysis of Highly Cited Studies

Citation analysis is an important part of bibliometric research. The citation rate of an article reflects its influence in the field to a certain extent. Table 2 lists the top 10 most-cited papers in the study on the rehabilitation of hip fracture in the elderly. All these studies were published between 2001 and 2005, and all of them were published more than 200 times. Among them, eight were original articles, and two were systematic reviews. Specifically, an original article entitled “Reducing delirium after hip fracture: A randomized trial (19)” published in the *Journal of the American Geriatrics Society* has been cited 754 times and is the top-cited paper in the field. The second and third most-cited papers were published by Braithwaite et al. (20) and Leibson (21), respectively, which are the study of morbidity, mortality, and cost of hip fracture.

**Table 2:** The top 10 most-cited papers in the study on rehabilitation of hip fracture in the elderly.

**Title**	**Journal**	**Autor**	**Publication year**	**Citations**
Reducing delirium after hip fracture: A randomized trial	Journal of the American Geriatrics Society	Marcantonio ER	2001	754
Estimating hip fracture morbidity, mortality and costs	Journal of General Internal Medicine	Braithwaite RS	2003	568
Mortality, disability, and nursing home use for persons with and without hip fracture: A population-based study	Journal of the American Geriatrics Society	Leibson CL	2002	445
Association of timing of surgery for hip fracture and patient outcomes	JAMA	Orosz GM	2004	370
Efficacy of a comprehensive geriatric intervention in older patients hospitalized for hip fracture: A randomized, controlled trial	Journal of the American Geriatrics Society	Vidan M	2005	324
The impact of post-operative pain on outcomes following hip fracture	Pain	Morrison RS	2003	322
Mortality and locomotion 6 months after hospitalization for hip fracture - Risk factors and risk-adjusted hospital outcomes	JAMA	Hannan EL	2001	309
Randomized controlled trial to investigate influ–ence of the fluid challenge on duration of hospital stay and perioperative morbidity in patients with hip fractures	British Journal of Anesthesia	Venn R	2002	303
Economic implications of hip fracture: Health service use, institutional care and cost in Canada	Osteoporosis International	Wiktorowicz ME	2001	286
Effects of extended outpatient rehabilitation after hip fracture - A randomized controlled trial	JAMA	Binder EF	2004	237

### Analysis of Authors and Co-cited Authors

**Figure 5:**
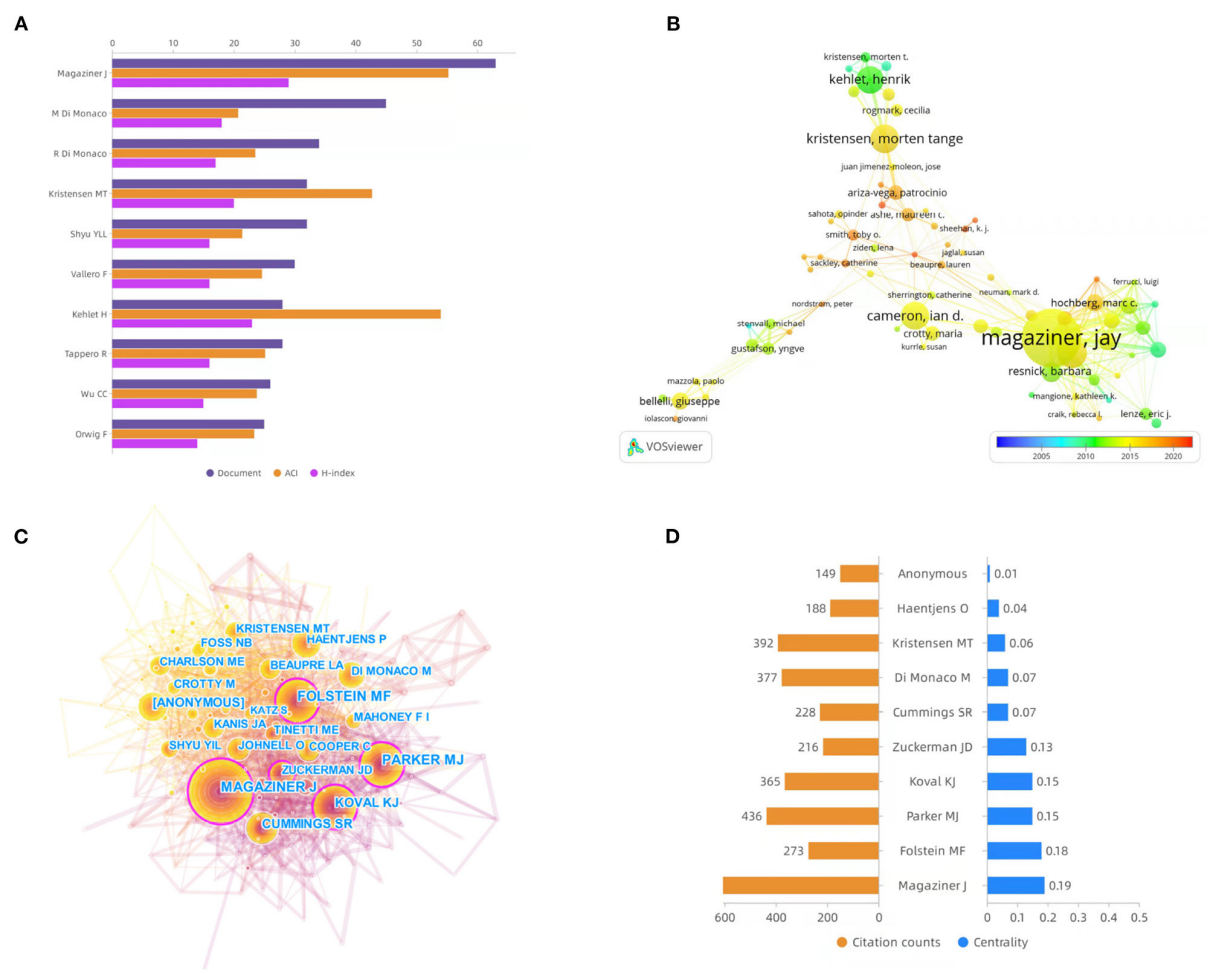
**(A)** The publication counts, H-index, and ACI of the top 10 most prolific authors. **(B)** Overlay visualization map of author coauthorship analysis generated by VOSviewer software. **(C)** Visualization map of author co-citation analysis by using CiteSpace software. **(D)** The citation counts and centrality of the top 10 most co-cited authors.

The number of articles published by a researcher represents the level of contribution and activity in the field. As shown in Figure 5A, Magaziner ranked first in the number of documents, H-index, and ACI. In terms of other data, M. Di Monaco ranked second in the number of documents, while Kehlet ranked second in the H-index and ACI. In Figure 5B, an overlay visualization map of author coauthorship analysis was generated by VOSviewer software. They created several research clusters, each radiated by one or two core authors, such as Magaziner, Kehlet, and Kristensen. In general, there are very few connections between different clusters, indicating that collaboration in this area is not well developed. Author co-citation analysis is usually used to reveal the key authors in the co-citation network in a certain field. Generally, authors who are frequently cited have greater influence in the field. As shown in Figure 5C, nodes with centrality greater than 0.1 are circled in purple. The larger the node is, the more times the author is referenced. The top three most co-cited authors are Magaziner, Parker, and Kristensen. The top three authors for centrality are Magaziner, Folstein, and Parker. The citation counts and centrality of the top 10 most co-cited authors are shown in Figure 5D.

### Analysis of Keywords

**Figure 6:**
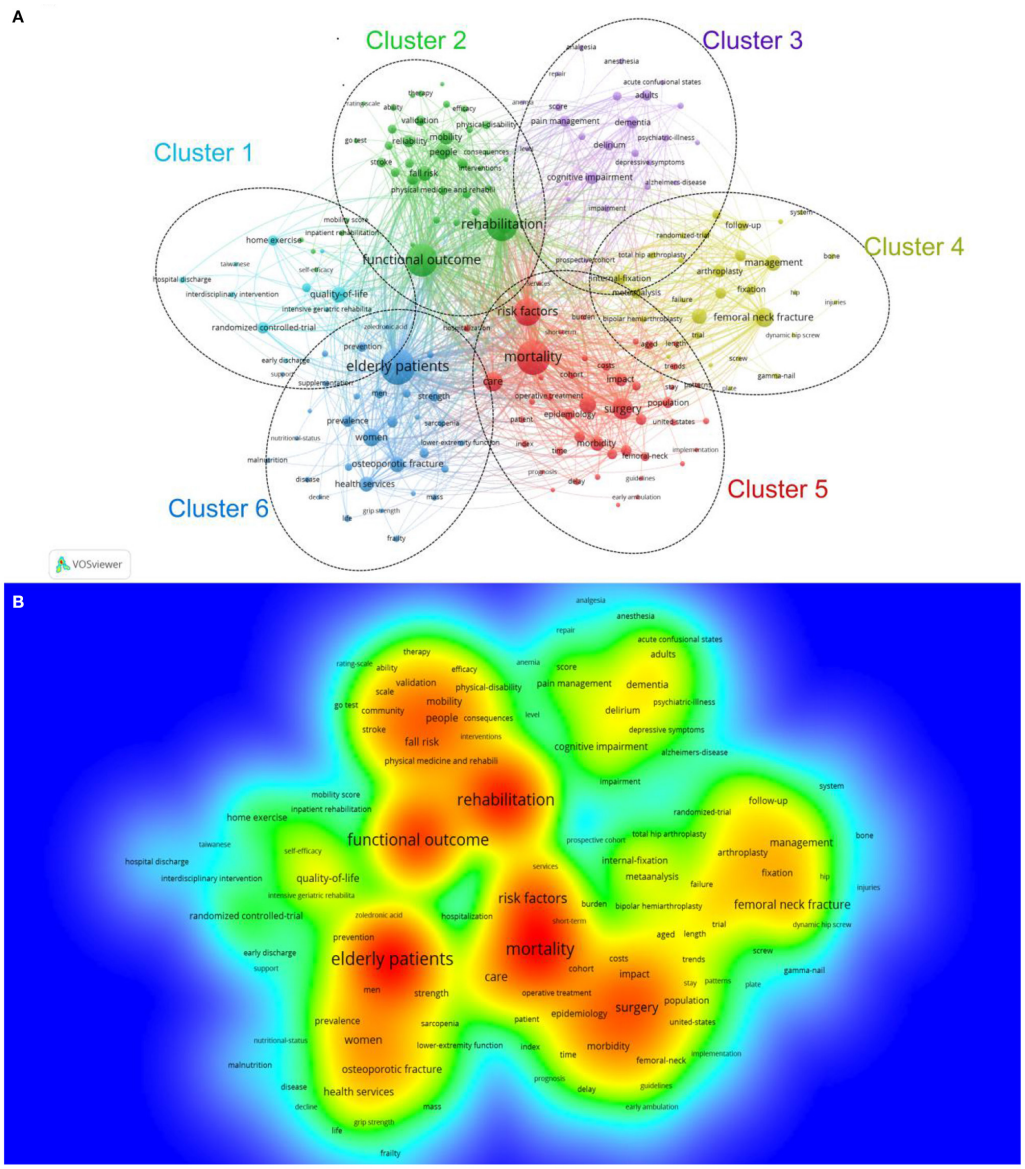
**(A)** Network visualization of the keyword co-occurrence analysis by VOS viewer. **(B)** The density map of keywords.

A total of 2,075 keywords were extracted from the 1,724 articles and analyzed by VOSviewer. We performed clustering analysis of these co-occurrence keywords. As shown in Figure 6A, all of them could be classified into five clusters: Cluster 1 (quality of life study), Cluster 2 (rehabilitation and outcomes study), Cluster 3 (cognitive impairment study), Cluster 4 (operative approaches study), Cluster 5 (mortality study), and Cluster 6 (osteoporosis study). These clusters showed the most prominent topics in the study on rehabilitation of hip fracture in the elderly thus far. As shown in Figure 6B, a density visualization map was generated for keywords with a co-occurrence greater than 10 times, which includes 608 keywords in the map.

## Discussion

In this study, we used bibliometrics and visual analysis to study the global research trends of postoperative hip fracture rehabilitation in terms of publications, contributing countries, institutions, journals, and authors. In the past two decades, research on the postoperative rehabilitation of elderly hip fractures has been increasing year by year. In particular, the number of articles published has continued to increase in the past 5 years. In 2021, 183 articles were published, which is a historical peak. According to the available knowledge, many factors contribute to the rapid progression of research on the rehabilitation of hip fracture in the elderly. As the world's population ages, the incidence of hip fracture in the elderly is increasing. The incidence of hip fractures in China in patients over 55 years old was 136.65 per 100,000 people in 2016 (22). The large patient population has increased focus in the field. It is foreseeable that the literature in this field will continue to grow in future.

According to the analysis of the number of articles published by different countries, the USA publishes the most relevant documents in the world. The USA accounted for more than one-fifth of the documents from the top 10 countries, reflecting its strong research capacity and focus on the development of the field. It is worth noting that China has published the largest number of documents in the past 5 years. This shows that China is increasing attention on the research progress in this field.

For the research institutions, the most published and highlighted ACI institutions were Tel Aviv University and the University of Sydney, respectively. Such results could be attributed to the strong academic atmosphere and strong scientific research foundation of the two universities. Researchers can focus on these countries and institutions obtain timely information on research related to the postoperative rehabilitation of hip fractures. In the cooperation network of institutions, it is found that the cooperation of institutions is mainly distributed in European and American countries. China has a large patient population, but there is a lack of interinstitutional cooperation. In terms of funding support, five of the top 10 sources were from the USA. The USA has a strong economy that produces much research.

The *Injury: International Journal of the Care of the Injured* published the most articles/reviews. *Injury* was founded in 1969 and is an international journal dealing with all aspects of trauma care and accident surgery (23). The *Journal of the American Geriatrics Society* ranks first in impact factor, ACI, and H-index. The *Journal of the American Geriatrics Society* is an important journal for clinical aging research. It provides a diverse, interprofessional community of healthcare professionals with the latest insights into geriatric education, clinical practice, and public policy (24). Additionally, in the co-citation network, *Disability and Rehabilitation* had the largest TLS, followed by the *Journal of the American Geriatrics Society* and the *Archives of Physical Medicine and Rehabilitation*. Therefore, we should pay attention to the dynamics of these journals in future research.

Dual-map overlay analysis is helpful to understand the flow of research hot spots among different disciplines. As shown in Figure 4C, the citing journals are located on the left, and the cited journals are located on the right. The arrow points to the citation paths showing that there are four core routes, including two green and two pink. The green paths indicate that documents published in Medicine/Medical/Clinical journals usually cited documents published in journals belonging to Sports/Rehabilitation, Dermatology/Dentistry/Surgery, and Health/Nursing/Medicine. The pink paths imply that the majority of papers published in the journals of Neurology/Sports/ Ophthalmology are likely to be biased to cite papers published in journals within Sports/Rehabilitation, Dermatology/Dentistry/Surgery, and Health/Nursing/medicine.

From our highly cited studies analysis, an original article entitled “Reducing delirium after hip fracture: A randomized trial” and published in the *Journal of the American Geriatrics Society* is the top-cited paper in the field. This article confirms that proactive geriatric consultation was successfully implemented with good adherence after hip fracture repair. Geriatric consultation reduced delirium by over one-third and reduced severe delirium by over one-half (19). The second and third most-cited papers were published by Braithwaite et al. (20) and Leibson (21), which are the study of morbidity, mortality, and cost of hip fracture. Notably, three of the top 10 were from the top journal *JAMA*. The authors are Orosz, Hannan, and Binder. Orosz's publication of the association of timing of surgery for hip fracture and patient outcomes is a major academic achievement. This study recommended that early surgery was associated with reduced pain and length of stay and probably major complications among patients who were medically stable at admission. Patients with hip fracture who are medically stable should receive early surgery when possible (25). Hannan's research argues that mortality and functional status should ideally be considered both together and individually to distinguish effects limited to one or the other outcome (26). Binder's research indicated that extended outpatient rehabilitation can improve physical function and the quality of life, and reduce disability compared with low-intensity home exercise (27).

In terms of author analysis, influential authors include Magaziner, Kehlet, Parker, and Kristensen. As mentioned above, Magaziner's publication of a randomized controlled trial of postoperative delirium after hip fracture is a major academic achievement (19). Kehlet is a famous surgeon from the University of Copenhagen, Denmark, who first proposed the concept of fast-track surgery (FTS) (28). Under his concept guidance, FTS has been rapidly developed. It has been applied to elderly patients with hip fracture, which has not only shortened the hospital stay but also reduced the mortality of patients (29). Parker MJ's major research contribution was to predict the risk of hip fracture after surgery. He has developed scoring systems for predicting the risk of hip fracture (30). Kristensen focused on the postoperative function of hip fractures. He published studies on factors influencing postoperative rehabilitation of hip fractures (31, 32).

In bibliometric analysis, frequently appearing keywords are often used to identify major and hot topics in a research field by generating a co-occurrence network map. In the present study, a total of 2,075 keywords were extracted from the 1,724 articles and analyzed by VOSviewer. Eventually, all of them could be classified into five clusters: Cluster 1: quality of life study. Improving the quality of life is a challenge for the postoperative rehabilitation of hip fracture in elderly people. Active rehabilitation after hip fracture in the elderly is helpful to improve the quality of life (3). Cluster 2: rehabilitation and outcomes study. In general, the earlier the intervention, the better the prognosis. As mentioned above, the concept of FTS has been applied to hip fractures. It has not only shortened the hospital stay but also reduced the mortality of patients (29). However, it is still being explored and needs constant improvement. Cluster 3: cognitive impairment study. Cognitive impairment is common among older adults with hip fractures. The estimated prevalence of cognitive impairment among older adults with hip fractures was 41.8% (33). It is helpful to develop special rehabilitation programs for patients with cognitive dysfunction (34). However, most rehabilitation services for individuals who currently sustain a hip fracture are not designed to meet the complex needs of those who also have cognitive impairment (35). Cluster 4: operative approaches study. The choice of surgical method has a certain influence on the postoperative recovery of patients. Generally, femoral neck fractures are treated with joint replacement or cannulated screw fixation, while intertrochanteric fractures are treated with intramedullary nailing. In the treatment of femoral neck fractures, patients treated with joint replacement achieved earlier time out of bed than those treated with internal fixation (36). Cluster 5: mortality study. Postoperative rehabilitation intervention is helpful to reduce the mortality of hip fracture (37). Cluster 6: osteoporosis study. Hip fracture is the most serious consequence of falling in older people with osteoporosis. Therefore, the treatment of osteoporosis should be considered during the implementation of rehabilitation programs (38). These clusters showed the most prominent topics in the research of postoperative rehabilitation of hip fracture in elderly people thus far.

### Limitations

The present study had some limitations inherent in bibliometrics. First, this study only included the WoSCC database, and no other databases, so some important studies may be omitted. Second, only literature published in English was included in the retrieval of Web of Science database in this study, which may lead to bias in the research results. In addition, for some recently published high-quality papers, a short publication time leads to a low citation frequency, which may affect the total citation frequency and H-index of the literature. This produces errors in the overall evaluation of the literature quality level.

## Conclusion

In the past 20 years, the number of published studies on the rehabilitation of hip fracture in the elderly has exhibited an overall upward trend. There will be an increasing number of publications on the research of postoperative rehabilitation of hip fracture in elderly people, with the United States remaining ahead in this field. The international cooperation map among relevant countries/regions indicated that the USA collaborated most closely with Canada and China. The University of Maryland and Professor Marcantonio were the most prolific institution and influential author, respectively. *Injury: International Journal of the Care of the Injured* was the most productive journal concerning the research of postoperative rehabilitation of hip fracture in elderly people. The keyword co-occurrence analysis identified six clusters: quality of life study, rehabilitation and outcomes study, cognitive impairment study, operative approaches study, mortality study, and osteoporosis study. Overall, our findings could offer practical sources for scholars to understand the current status and trend of studies on rehabilitation of hip fracture in the elderly and provide references and suggestions for the development of related research in future.

## Data Availability Statement

The original contributions presented in the study are included in the article/Supplementary Material, further inquiries can be directed to the corresponding author/s.

## Author Contributions

YL, QW, and BW designed the study. LH and ZL collected the data. LH, ZL, and ZW analyzed the data and drafted the manuscript. LH, ZW, MH, and HX revised and approved the final version of the manuscript. All authors contributed to the article and approved the submitted version.

## Funding

This study was sponsored by the Foundations of the Guangdong Research Institute for Orthopedics & Traumatology of Chinese Medicine (GYH202101-02) and the Guangdong Provincial Administration of Chinese Medicine Research Project (No. 20201168).

## Conflict of Interest

The authors declare that the research was conducted in the absence of any commercial or financial relationships that could be construed as a potential conflict of interest.

## Publisher's Note

All claims expressed in this article are solely those of the authors and do not necessarily represent those of their affiliated organizations, or those of the publisher, the editors and the reviewers. Any product that may be evaluated in this article, or claim that may be made by its manufacturer, is not guaranteed or endorsed by the publisher.
