# Supplementary material for: Mapping Knowledge Structure and Themes Trends of Post-operative Rehabilitation of Hip Fractures in the Elderly: A Bibliometrics and Visualization Study
Source: Front Surg. 2022 May 3;9:881555. doi: 10.3389/fsurg.2022.881555 (PMC9110792; doi:10.3389/fsurg.2022.881555)
Supplement: Supplementary file 1 [file Table_1.DOCX]

| **Supplemental Table 1: Top 10 journals in the study on rehabilitation of hip fracture in the elderly ranked by the publication number.** | | | | |
| --- | --- | --- | --- | --- |
| Countries/regions | Documents | Citations | ACI | Ratio |
| USA | 354 | 13786 | 38.94 | 20.65% |
| China | 161 | 1204 | 7.48 | 9.39% |
| Italy | 146 | 3231 | 22.13 | 8.52% |
| England | 144 | 3715 | 25.80 | 8.40% |
| Canada | 123 | 3751 | 30.50 | 7.18% |
| Australia | 107 | 2934 | 27.42 | 6.24% |
| Israel | 89 | 1506 | 16.92 | 5.19% |
| Spain | 85 | 1868 | 21.98 | 4.96% |
| Denmark | 83 | 2666 | 32.12 | 4.84% |
| Sweden | 83 | 2792 | 33.64 | 4.84% |
